# P-2021. How Have COVID-19 Treatment Guidelines and the Scientific Evidence Evolved throughout the Pandemic and Endemic Eras?

**DOI:** 10.1093/ofid/ofae631.2178

**Published:** 2025-01-29

**Authors:** Essy Mozaffari, Michele Bartoletti, Alpesh N Amin, Yohei Doi, Yohei Doi, Paul Loubet, Christina G Rivera, Michael Roshon, Aaditya Rawal, Emily Kaiser, Maria Vutcovici Nicolae, Olena Polyakova, Thomas F Oppelt, Mel Chiang, Paul E Sax, Andre Kalil

**Affiliations:** Gilead Sciences, Foster, California; Department of Biomedical Sciences, Humanitas University, Pieve Emanuele (Italy); IRCCS Humanitas Research Hospital, Rozzano (Italy), Rozzano, Lombardia, Italy; University of California, Irvine, Orange, California; Fujita Health University, Aichi, Aichi, Japan; Fujita Health University, Aichi, Aichi, Japan; CHU de Nîmes, Nimes, Languedoc-Roussillon, France; Mayo Clinic, Rochester, Minnesota; CommonSpirit Health, Mountain Region, Colorado Springs, Colorado; Costello Medical Inc., Boston, Massachusetts; Costello Medical, Boston, Massachusetts; Certara Canada, Montreal, Quebec, Canada; Certara, Montreal, Quebec, Canada; Gilead Sciences, Inc, Foster City, California; Gilead Sciences, Foster, California; Brigham and Women’s Hospital; Harvard Medical School, Boston, MA; University of Nebraska Medical Center

## Abstract

**Background:**

With progressive understanding of the natural history of COVID-19 and accumulation of knowledge on clinical management, treatment guidelines recommended several options including remdesivir (RDV), a broad-spectrum antiviral. Given the evolving nature of COVID-19, it is critical to capture the totality of scientific evidence to inform clinical decision making. We conducted a systematic literature review (SLR) to summarize the mortality endpoint for RDV among hospitalized adults throughout COVID-19 eras and contrasted with evidence informing treatment recommendations in clinical guidelines.

**Figure:**
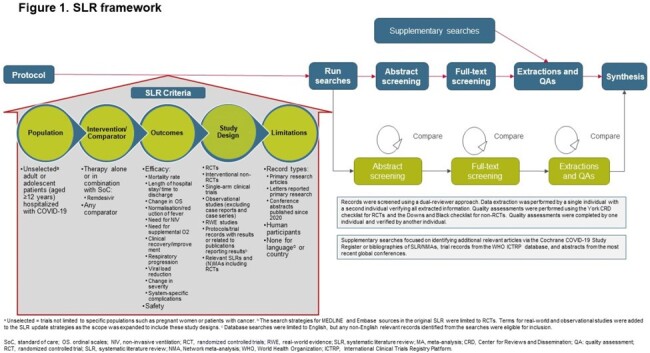

**Methods:**

We systematically searched MEDLINE, Embase and Cochrane Library databases for interventional and observational studies examining RDV efficacy. Clinical trial registries, Cochrane COVID-19 Study Register, and conference abstracts were hand-searched (Fig1). Case reports and case studies were excluded. A review of most recent RDV recommendations across guidelines was conducted.

**Figure:**
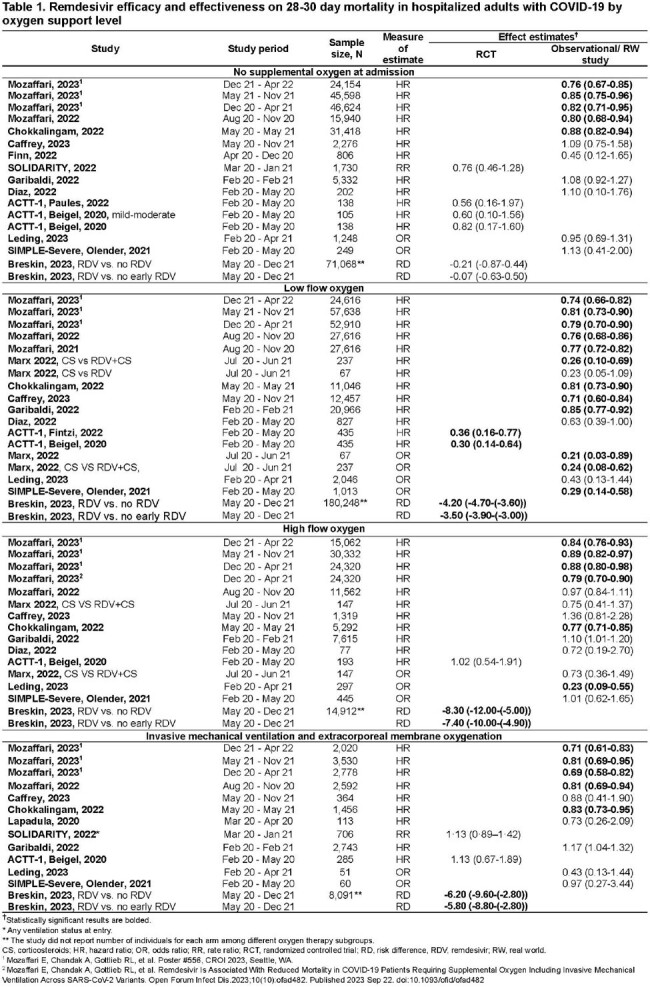

**Results:**

From Dec ’19 to Dec ’22, 123 relevant publications were identified. In ’23, the evidence grew significantly, with 70 new publications. In total, 193 publications were evaluated, and 122 unique studies were included. While early randomized clinical trials (RCT) and small sample size observational studies did not universally demonstrate a significant difference in mortality in all severity groups of RDV-treated patients, real-world studies with larger sample sizes showed a significant impact across disease severity levels, regardless of COVID-19 era (Table1). Guideline recommendations for COVID-19 treatment with RDV are based on early RCTs (Table2) and most have not been updated (Fig2).

**Figure:**
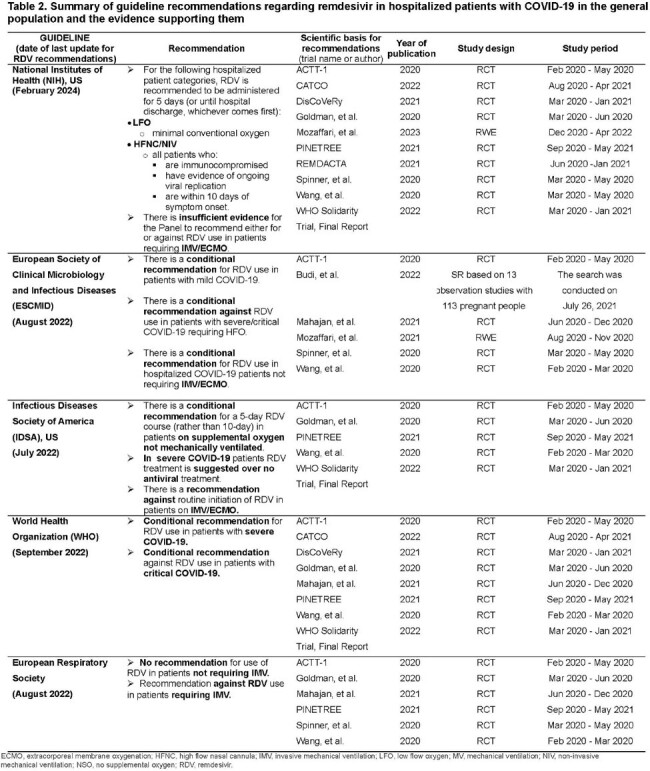

**Conclusion:**

Our comprehensive evaluation of scientific literature indicates that evidence of RDV impact on mortality in hospitalized COVID-19 patients continued to grow and cover full range of disease severity. Guideline recommendations have not evolved in parallel which may explain differing recommendations in certain subgroups (e.g. IMV/ECMO). To assure that providers in the hospital setting are aware of and deploy evidence-based optimal care for patients with COVID-19, recommendations should rely on current evidence, including real-world data.

**Figure:**
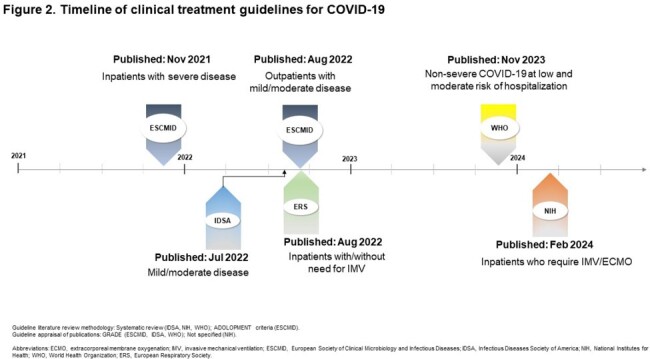

**Disclosures:**

Essy Mozaffari, PharmD, MPH, MBA, Gilead Sciences, Inc.: Employee|Gilead Sciences, Inc.: Stocks/Bonds (Public Company) Michele Bartoletti, MD, PhD, Advan pharma: Advisor/Consultant|Advan pharma: Honoraria|Biomereux: Honoraria|Gilead: Advisor/Consultant|Gilead: Honoraria|Infectopharma: Advisor/Consultant|Msd: Advisor/Consultant|Msd: Grant/Research Support|Msd: Honoraria|Pfizer: Honoraria Alpesh N. Amin, MD, MBA, Alexion: Advisor/Consultant|Aseptiscope: Advisor/Consultant|AstraZeneca: Advisor/Consultant|Bayer: Advisor/Consultant|Dexcom: Advisor/Consultant|Eli Lilly: Advisor/Consultant|Ferring: Advisor/Consultant|Gilead: Advisor/Consultant|GSK: Advisor/Consultant|Heartrite: Advisor/Consultant|Nova Nordisk: Advisor/Consultant|Pfizer: Advisor/Consultant|Renibus: Advisor/Consultant|Reprieve: Advisor/Consultant|Salix: Advisor/Consultant|Seres: Advisor/Consultant|Spero: Advisor/Consultant Yohei Doi, MD, PhD, bioMerieux: Lecture fees|Entasis: Grant/Research Support|Fujifilm: Advisor/Consultant|Gilead Sciences: Advisor/Consultant|GSK: Advisor/Consultant|KANTO CHEMICAL CO.,INC.: Grant/Research Support|KANTO CHEMICAL CO.,INC.: Patent for genotyping kit|MeijiSeika Pharma: Advisor/Consultant|Moderna: Advisor/Consultant|MSD: Lecture fees|Pfizer: Advisor/Consultant|Shionogi & Co., Ltd.: Grant/Research Support|Shionogi & Co., Ltd.: Lecture fees Paul LOUBET, MD, PhD, Astrazeneca: Advisor/Consultant|Gilead: Advisor/Consultant|Moderna: Advisor/Consultant|Pfizer: Advisor/Consultant Christina G. Rivera (O'Connor), Pharm.D, Gilead Sciences: Board Member Michael Roshon, MD/PhD, MD/PhD, Gilead: Honoraria Thomas F. Oppelt, PharmD, BCPS, Gilead Sciences, Inc: I am an employee of Gilead Sciences, Inc|Gilead Sciences, Inc: Stocks/Bonds (Public Company) Mel Chiang, Ph.D., Gilead Sciences: I am an employee of Gilead Sciences|Gilead Sciences: Stocks/Bonds (Public Company)

